# Odontoblast control of dental pulp inflammation triggered by cariogenic bacteria

**DOI:** 10.3389/fphys.2013.00326

**Published:** 2013-11-11

**Authors:** Jean-Christophe Farges, Brigitte Alliot-Licht, Caroline Baudouin, Philippe Msika, Françoise Bleicher, Florence Carrouel

**Affiliations:** ^1^Institut de Génomique Fonctionnelle de Lyon, Equipe Physiopathologie des Odontoblastes, Ecole Normale Supérieure de Lyon, CNRS UMR5242Lyon, France; ^2^Faculté d'Odontologie, Université de Lyon, Université Lyon 1Lyon, France; ^3^Hospices Civils de Lyon, Service de Consultations et Traitements DentairesLyon, France; ^4^Faculté d'Odontologie, INSERM UMR1064, Centre de Recherche en Transplantation et Immunologie, Université de NantesNantes, France; ^5^Laboratoires ExpanscienceEpernon, France

**Keywords:** human tooth, healing, dentine, inflammation, repair, odontoblast, caries, dental pulp

Inflammation is part of the normal protective immune response of the host to tissue infection. It promotes the recruitment of circulating immunocompetent blood cells and their migration through the endothelial barrier to gain access to the damaged site and eliminate injurious pathogens. If kept uncontrolled, inflammation can result in a wide range of acute, chronic, and systemic inflammatory disorders (Serhan and Petasis, 2011). Therefore, higher organisms have evolved protective mechanisms to ensure the inflammatory response is resolved in a specific time-limited manner (Serhan et al., 2008). Resolution of inflammation requires the elimination of injurious agents and the removal of pro-inflammatory mediators that initiate host defense against microbial invasion. In addition, anti-inflammatory agents including steroids, IL-1 receptor antagonist, soluble TNF receptor, interleukin-10 (IL-10), nitric oxide (NO), heme oxygenase-1, as well as regulatory T lymphocytes (Tregs), are produced to limit tissue damage and promote return to homeostasis (Gilroy et al., 2004; Eming et al., 2007; Blancou and Anegon, 2010; Buckley et al., 2013). Recent studies have revealed that endogenous lipid mediators, such as lipoxins and resolvins, synthesized locally during the inflammatory phase, stimulate cellular and molecular events that define the resolution of inflammation and repair (Serhan and Petasis, 2011). Complete cessation of inflammation is thus an active, multifactorial and highly orchestrated process (Uddin and Levy, 2011; Rius et al., 2012).

A major cause of inflammation in human dental pulp is the presence, in the affected dentine, of the oral bacteria responsible for carious lesion development (Love and Jenkinson, 2002). Pulp inflammation accompanies the host's innate and adaptive immune responses to these bacteria and/or to their components released during bacterial growth that diffuse to the pulp through dentine tubules. It generally dampens after pathogen removal by the dental practitioner and neutralization of diffusing components by the pulp immune system, which both decrease the production of pro-inflammatory mediators (Hahn and Liewehr, 2007). However, in cases of important dentine damage, pulp inflammation does not resolve completely but becomes chronic with moderate inflammatory infiltrate, collagenous fibrosis and premature tissue aging, and sometimes leads to pulp necrosis and dental abscess development. These evolutions induce permanent loss of normal tissue function and reduction of pulp defense capacities to future injuries. Conversely, cessation of pulp inflammation generally induces the re-establishment of homeostasis and accurate tissue healing characterized by maintenance of pulp vitality, absence of inflammatory infiltrate and fibrosis, and formation of a barrier of reactionary dentine by surviving original odontoblasts and/or reparative dentine by newly differentiated odontoblast-like cells (Lesot et al., 1994). Dentine neoformation moves the pulp tissue away from the dentine injury and the crown filling biomaterial, thus reducing the risk of permanent irritation by external chemical or bacterial agents. In the light of what happens in other healing tissues, it is reasonable to speculate that the more rapidly dentine neoformation is initiated, the quicker pulp homeostasis and health are re-established.

Pulp inflammation resulting from carious lesions is characterized by a strong increase in the production of pro-inflammatory cytokines including TNF-α, IFN-γ, IL-1β, IL-6, CXCL8, and IL-18. Interestingly, IL-10, a cytokine that plays a central role in limiting host immune response to pathogens by promoting the development of Tregs is also upregulated (Farges et al., 2011). An increase in the production of NO, a free radical anti-inflammatory at high concentration (Connelly et al., 2001), is also observed in bacteria-challenged, inflamed dental pulps (Di Nardo Di Maio et al., 2004; Korkmaz et al., 2011). The role of NO in this context remains unclear but experiments have suggested that, besides its well-known roles in vascular tone and nociceptive input modulation, it may be implicated in dental pulp healing by promoting odontoblast-like cell differentiation and subsequent formation of reparative dentine (Mei et al., 2007; Yasuhara et al., 2007). Recently, special attention was paid to lipopolysaccharide-binding protein (LBP), an acute-phase protein known to attenuate pro-inflammatory cytokine production by macrophages activated with bacterial components. LBP was shown to prevent binding of several bacterial cell wall components including lipopolysaccharides, lipoteichoic acids, lipopeptides, and peptidoglycan to host cells (Schumann, 2011; Lee et al., 2012). Interestingly, it was found to transfer lipopolysaccharides to high-density lipoproteins in the plasma for neutralization (Wurfel et al., 1995). We recently detected LBP synthesis and accumulation in bacteria-challenged inflamed pulp, whereas this protein was not found in healthy pulp (Carrouel et al., 2013). We proposed this molecule is involved in the neutralization of bacterial components before they gain access to pulp cells, thus limiting activation of the pulp immune system and the associated inflammatory response. Despite these important findings, the effects of IL-10, NO, and LBP in the control of dental pulp inflammation and promotion of pulp healing remain largely unknown. Studies are thus warranted to evaluate their importance in these processes and elucidate their putative therapeutic potential.

**Figure 1:**
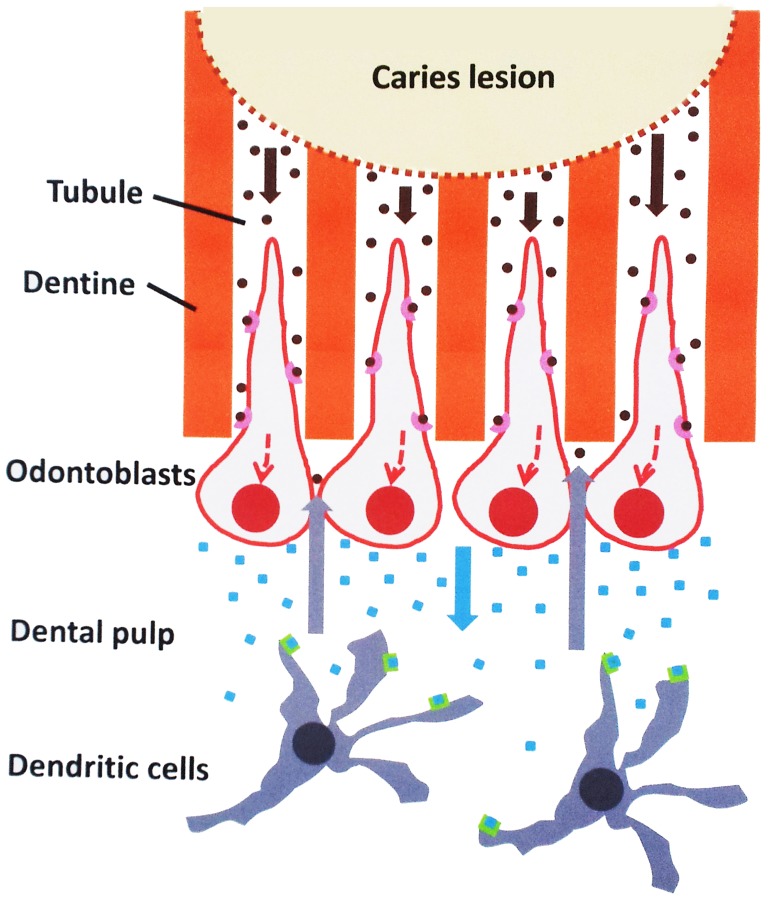
**Possible role of odontoblasts in the initiation of the dental pulp immune response to cariogenic bacteria**. Oral bacteria that degrade dentine during carious lesions release pathogen-associated molecular patterns (PAMPs; brown dots) that may diffuse through the tubules to the odontoblast layer (brown arrows). Here, they are recognized by specific pathogen recognition receptors (pink cups) present at the odontoblast surface. Activation of specific intracellular pathways (dotted red lines) leads to the production of pro-inflammatory mediators including chemokines (blue squares) secreted at the opposite pole of the cell. These chemokines diffuse in the subodontoblast pulp area (blue arrow) and, upon binding to specific receptors (green boxes) attract antigen-presenting immature dendritic cells that ensure tissue immunosurveillance. These cells migrate to the odontoblast layer (gray arrows) to capture PAMPs arriving at the tubule pulpal end and develop the immune response and the associated inflammation.

Bacterial components that trigger innate immune responses are mostly represented by a limited number of evolutionary-conserved, structural motifs found in a wide range of microbes and called Pathogen-Associated Molecular Patterns (PAMPs) (Beutler, 2009). PAMP recognition (or sensing) is mediated by a set of specific germline-encoded host receptors referred to as Pattern Recognition Receptors (PRRs). PRRs are mainly localized at the cell surface or are present in the cytosol or in endosomes (Takeuchi and Akira, 2010). Owing to their specific localization at the pulp-dentin interface and the entrapment of their long cell processes in dentine tubules, odontoblasts are the first cells challenged by intradentinal PAMPs and it's been proposed they are involved in the PAMP recognition process (Figure 1). Immunolocalization of PRRs of the Toll-like receptor family in the odontoblast cell membrane (TLR2 and TLR4), as well as their activation in odontoblasts and odontoblast-like cells *in vitro* by specific PAMPs, argue in favor of this hypothesis (Durand et al., 2006; Veerayutthwilai et al., 2007). Odontoblasts thus constitute, in the tooth, the first line of defense for the host and are suspected to be involved in the initiation, development and maintenance of the pulp immune and inflammatory responses to dentine-invading pathogens. Studies have shown that upon TLR2 stimulation odontoblasts activate specific intracellular signaling pathways involving NF-κB and p38 MAP kinase (Carrouel et al., 2013). This activation leads to odontoblast down-regulation of dentine formation, production of pro-inflammatory molecules including interleukin-6 (IL-6) and CCL2, CXCL1, CXCL2, and CXCL8 chemokines, as well as immature dendritic cell accumulation into the odontoblast layer close to the carious dentine (Farges et al., 2009). The immunosuppressive cytokine IL-10 is up-regulated, suggesting odontoblasts may participate in limiting the inflammatory process in bacteria-challenged pulps (Farges et al., 2011). Up-regulation of LBP *in vitro* in PAMP-stimulated odontoblast-like cells (unpublished results) and *in vivo* in odontoblasts challenged by intradentinal cariogenic bacteria (Carrouel et al., 2013) could also contribute to shorten pulp inflammation duration. It is currently unknown whether these inflammation-dampening effects, by modulating specific intracellular signaling pathways, allow odontoblasts to recover their dentinogenic functions, an important event for dentine neoformation at the pulp-lesion interface and pulp healing.

Resolution of inflammation is essential to maintain host health and several families of specialized “pro-resolving” local mediators (SPMs) including lipoxins, resolvins, protectins, and maresins have been involved in the clearance and regulation of inflammatory exudates to restore tissue homeostasis (Serhan et al., 2008). SPMs are biosynthesized from ω-3 poly-unsaturated fatty acids and provide local control over the execution of an inflammatory response toward resolution. In particular, they inhibit NF-κB and MAP kinase signaling pathways and pro-inflammatory cytokine production (Serhan and Petasis, 2011; Uddin and Levy, 2011). In spite of the importance of these lipid mediators, no studies have been published on their production in the inflamed dental pulp.

In conclusion, we propose that identifying odontoblast molecules and mechanisms involved in the cessation of dental pulp inflammation is a crucial step for developing natural, host-derived agents able that promote rapid return to dental pulp homeostasis and health after pathogens are removed from caries-affected dental tissues.
